# Epidemiology and ecology of the sylvatic cycle of African Swine Fever Virus in Kenya

**DOI:** 10.1016/j.virusres.2024.199434

**Published:** 2024-07-19

**Authors:** Vincent Obanda, Mercy Akinyi, Edward King'ori, Ruth Nyakundi, Griphin Ochola, Purity Oreng, Kevin Mugambi, Grace Mwihaki Waiguchu, Mary Chege, William Rosenbaum, Erik Bovinder Ylitalo, Anne Tuiskunen Bäck, Lisa Pettersson, Opanda Silvanos Mukunzi, Bernard Agwanda, Susanna Stenberg-Lewerin, Olivia Wesula Lwande

**Affiliations:** a Veterinary Science and Laboratories Department, Wildlife Research and Training Institute, P.O Box 842-20117, Naivasha, Kenya; b Institute of Primate Research, P.O. Box 24481 Karen 00502, Nairobi, Kenya; c Veterinary Services Department, Kenya Wildlife Service, P.O. Box 54582 00200, Nairobi, Kenya; d Center for Savannah, Arid and Semi-Arid Ecosystems, Wildlife Research and Training Institute, Tsavo, P.O Box 842-20117, Naivasha, Kenya; e Department of Medical Biosciences, Clinical Genomics, 901 87, Umeå University, Umeå, Sweden; f Department of Diagnostics and Intervention, Umeå University, 901 87, Umeå, Sweden; g Department of Clinical Microbiology, Umeå University, 901 85, Umeå, Sweden; h Centre for Virus Research, Kenya Medical Research Training Institute, P.O. BOX 54628-00200, Nairobi, Kenya; i Zoology Department, National Museums of Kenya, P.O. BOX 40658- 00100, Nairobi, Kenya; j Department of Animal Biosciences, Swedish University of Agricultural Sciences, P.O Box 7023 75007, Uppsala, Sweden; k Umeå Centre for Microbiology Research, Umeå University, 901 87, Umeå, Sweden

**Keywords:** Tick-borne diseases, Microbial community, Food security, Soft ticks, *Ornithodoros moubata*, *Argasid* ticks

## Abstract

African Swine Fever (ASF) is caused by a DNA virus (AFSV) maintained and transmitted by the *Argasid* ticks. The re-emergence of the disease in Africa coupled with its rapid spread globally is a threat to the pig industry, food security and livelihoods. The ecology and epidemiology of the ASFV sylvatic cycle, especially in the face of changing land use and land cover, further compounds the menace and impacts of this disease in Kenya. The study aimed to determine the occurrence and distribution of ASFV seroprevalence in warthog populations, the tick vectors and extent of tick infestation of warthog burrows, and the genotypes of ASFV in soft ticks in Kenya. Warthogs from different parts of Kenya were captured and venous blood was centrifuged to harvest sera. Warthog burrows were examined for their conditions and to extract ticks. Sera were analyzed for antibodies against ASFV using a commercial ELISA kit coated with p32 ASFV recombinant protein. Ticks were pooled, DNA extracted and the p72 gene of the ASFV was amplified by qPCR and conventional PCR. The overall seroprevalence of ASFV in warthogs was 87.5 %. A total of 228 warthog burrows were examined and 2154 argasid ticks were extracted from the burrows. Tick pools from Kigio Farm and Lewa Wildlife Conservancies were ASFV-positive by qPCR and conventional PCR. ASFV was further confirmed by the Twist Comprehensive Viral Research Panel (TCVRP), which also identified the argasid ticks as *Ornithodoros porcinus*. The ticks were infected with virus genotype IX, and their occurrence overlaps with regions of previous ASF outbreaks in domestic pigs. Further, Viruses that could be tick endosymbionts/commensals or due to bloodmeal were detected in ticks by TCVRP; Porcine type-C oncovirus; *Pandoravirus neocaledonia; Choristoneura fumiferana granulovirus*; Enterobacteria phage p7; Leporid herpesvirus 4 isolate; 5; Human Lymphotropic virus; Human herpesvirus 5. In conclusion, our results suggest that infected *Ornithodoros* spp. seems to have a rich virome, which has not been explored but could be exploited to inform ASF control in Kenya. Further, the ecology of *Ornithodoros* spp. and burrow-use dynamics are complex and more studies are needed to understand these dynamics, specifically in the spread of ASFV at the interface of wild and domestic pigs. Further, our results provide evidence of genotype IX ASFV sylvatic cycle which through *O. porcinus* tick transmission has resulted in high exposure of adult common warthogs. Finally, the co-circulation of ASFV genotype IX in the same location with past ASF outbreaks in domestic pigs and presently in ticks brings to focus the role of the interface and ticks on virus transmission to pigs and warthogs.

## Highlights

• Detection of African Swine Fever Virus (ASFV) in *Ornithodoros porcinus* in Kenya.
• *Ornithodoros porcinus* is a vector of Genotype IX ASFV.
• Common warthogs have a high exposure to ASFV.
• Twist Comprehensive Viral Research Panel (TCVRP) can be used for detection of DNA viruses as well as barcoding of. tick species
• *Ornithodoros porcinus* seems to have a rich virome, which is yet to be explored but could be exploited to inform ASF control in Africa.

## Graphical abstract

**Figure:**
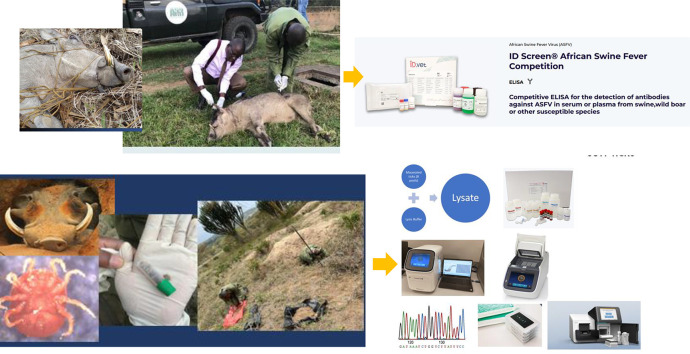

## 1 Introduction

The re-emergence and spread of the African swine fever virus (ASFV) is a threat to the global pig industry and causes major disruption in the socio-economics and livelihoods of communities in low-resource settings. The soft ticks of the *Ornithodoros* complex are the main reservoirs and vectors of this DNA virus (family *Asfaviridae*, genus *Asfivirus*) which causes up to 100 % case fatality in naïve domestic pigs (Barongo et al., 2015; Bisimwa et al., 2021; Jori and Bastos, 2009). The disease in pigs is characterized by haemorrhages, fever, bloody diarrhoea and disseminated intravascular coagulation leading to death within 1–7 days (Blome et al., 2013). The epidemiology and transmission of ASFV are complex and continue to be a challenge. For instance, the sylvatic cycle of ASFV revolves strictly around the warthog and the ticks. So far there is no evidence of the involvement of ticks in the domestic pig cycle, or in transmission of ASFV to bush pigs and giant forest hogs. ASFV has been isolated from a giant forest hog by (Heuschele and Coggins, 1965) with no link to ticks and limited contact with pigs. Moreover, bush pigs have been shown experimentally to be resistant to the virus (Anderson et al., 1998) but the situation has not been ascertained in nature. In the experimental study, the infected *O. moubata* ticks were able to transmit the virus to the domestic pigs leading to infection, however, the infected domestic pigs were not able to transmit the infection to in-contact bushpigs.

The ASF outbreaks in Kenya are thought to be due to direct pig-to-pig transmission (Thomas et al., 2016). The population of wild suids includes warthogs, bush pigs, and giant forest hogs, are widely distributed across Kenya and their grazing range overlaps with free-range domestic pigs. Although mainly driven by direct and indirect contacts between domestic pigs, indirect ASFV transmission could potentially occur at the interface with wildlife via contaminated water, soil, pasture, firewood and other environmental materials that may be brought in contact with domestic pigs at the homesteads (Okoth et al., 2013)

ASFV was first identified in Kenya in 1921 (Montgomery, 1921), but there is little detailed knowledge about the sylvatic cycle of the virus in Kenya (Abworo, 2012). Specifically, knowledge is sparse on the ecology and epidemiology, including the circulating genotypes, infection and exposure dynamics of wild suids, and tick-host-burrow interactions. Even though ASFV exposure may exceed 80 % in adult warthogs, they do not have detectable viremia (Thomson, 1985). It is suggested that since naïve young warthogs can maintain low viremia for several weeks (Anderson et al., 1998; Thomson et al., 1980), they are more critical to the sylvatic cycle as they transmit the virus to soft ticks within the burrows since the *argasid* soft ticks attach for a short period of time and feed rapidly (Boomker et al., 1991; Horak et al., 1988, 1983). Moreover the nymphs have also been found attached on warthogs outside burrow especially in situations where they may have not finished blood feeding (Boomker et al., 1991). The *Ornithodoros* spp. complex is the main tick reservoir of the virus, and despite transtadial and transovarial transmission of the virus in the tick (Plowright et al., 1970), the proportion of ASFV-positive ticks is usually less than 5.1 % (Netherton et al., 2019; Plowright et al., 1970), except in Tanzania where studies confirm positivity in 19 % of *Ornithodoros (O) porcinus (p) porcinus* and 15 % of *O. p. domesticus* and 18 % in the O. moubata complex group (Peter et al., 2021; Quembo et al., 2018). It is also observed that tick infestation of warthog burrows is variable, ranging between 30 % and 88 % (Netherton et al., 2019). The objective of this study was to address epidemiological and ecological gaps in the sylvatic cycle of ASFV in Kenya, which includes the occurrence and distribution of ASFV seroprevalence in warthog populations, the tick vectors, the extent of tick infestation of warthog burrows, and the genotypes of ASF virus in soft ticks collected from burrows in different study sites.

## 2 Materials and methods

### 2.1 Ethical considerations

The study protocol was approved by the Wildlife Research and Training Institute (WRTI), the state agency mandated to issue research permission in the wildlife sector, under the permit number WRTI-0143–01–22. The capture and sampling were carried out by the immobilization protocol of the Kenya Wildlife Service.

### 2.2 Study area

**Fig. 1:**
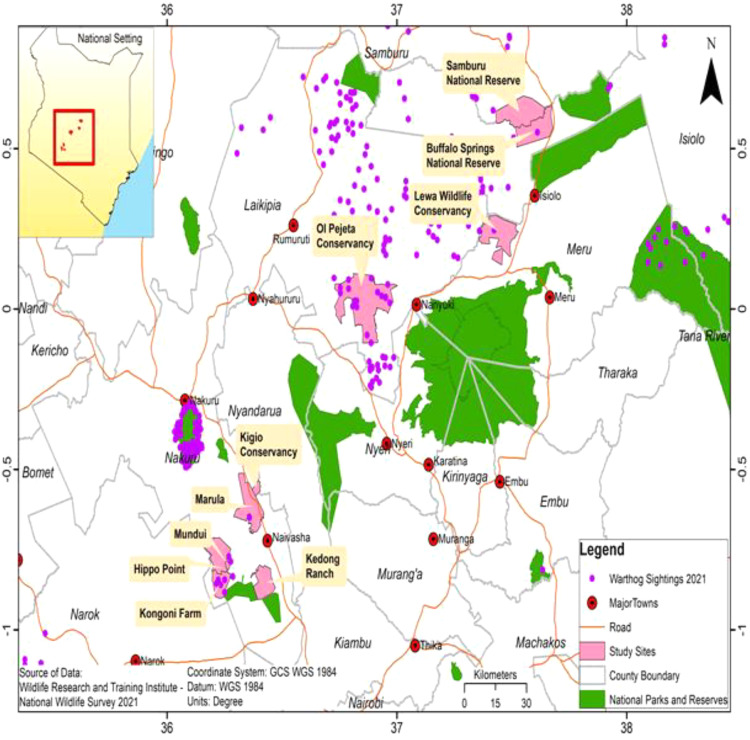
Map of study area showing locations of warthog populations, and sampling areas for ticks and burrows in Kenya.

Sampling of warthogs, tick extraction from burrows and burrow assessments were carried out in April, July, September and November of 2022. The study was carried out in several locations in Kenya, including Samburu National Reserve, Buffalo Springs National Reserve, Lewa Wildlife Conservancy, Ol Pejeta Conservancy, Kigio Conservancy, Marula Ranch, Mundui Farm, Kedong Ranch, Hippo Point and Kongoni Farm (Fig. 1). Wildlife and livestock (cattle, sheep, goats, camels) co-graze in these study areas. The study locations occur in the arid and semi-arid savanna ecosystems that sustain populations of common warthogs, among other wildlife. The warthog sighting dataset (Fig. 1) was sourced from the WRTI National Wildlife Census 2021, based on aerial surveillance. Layers of the Wildlife protected areas were sourced from Kenya Wildlife Service and Kenya Wildlife Conservancies Association. The GIS layers were analyzed and the map developed using the GIS software ArcMap 10.7 (2011)(“https://www.esri.com/news/arcnews/spring11articles/2011-esri-international-user-conference.html,” n.d.).

### 2.3 Sampling of warthogs

Warthogs were captured using standard animal capture nets erected in the bush. Warthogs become entangled in the nets and are physically restrained. About 10 ml of blood was drawn from the abdominal mesenteric vein into serum BD vacutainer® tubes, and placed in a cool box for a minimum of 4 h. Clear serum was pipetted into cryovials after a 15-minute spin in a centrifuge at 1500 *g*. The sera were stored in a freezer until processing.

### 2.4 Sampling of soft ticks from warthog burrows

Surveillance and detection of burrows in the expansive savanna were done by spotting raised soil mounds from the vehicle. The animals that dig out the burrow often pool the soil on one side of the burrow, which creates a raised mound. The burrows were sampled manually by use of a narrow-sized shovel fitted on a 1.5-meter pole. The walls of the burrow were scraped in 360° and the soil scooped out. The soil was placed on a clean polythene paper under sunlight, the soil spread out and the presence of ticks was noted by their movement. All ticks from each burrow per study location were placed in single/multiple vials and labelled with a specific number (Tables 2 and 3). The ticks were counted and recorded on-site.

**Table 1:** Prevalence of African Swine Fever virus in warthogs, number of sampled burrows and collected ticks from the burrows in different locations in Kenya.

S/N	Sampling site	Sample size	Positive warthogs	Negative warthogs	% Prevalence	No. of burrows	No. of ticks	Ticks per burrow	Average soil scoop per burrow
1	Hippo point	23	23	0	100	13	93	13.9	6
2	Samburu National Reserve	2	2	0	100	–	–		–
3	Kongoni	1	1	0	100	14	571	2.4	6
4	Marula	1	0	1	0	–	–		–
5	Ol Pejeta Conservancy	2	0	2	0	–	–	–	–
6	Buffalo Springs National Reserve	1	1	0	100	–	–	–	–
7	Mundui farm	5	4	1	80	4	137	2.9	6
8	Naivasha town	5	4	0	80	–	–		
9	Lewa Wildlife Conservancy	–	–	–	–	174	1258	13.8	6
10	Kigio Conservancy	–	–	–	–	13	95	13.6	6
11	Kedong	–	–	–	–	10	0		6
	**Overall**	**40**	**35**	**3**	**87.5**	**228**	**2154**	**10.5**

**Table 2:** Proportion of examined burrows and extracted ticks in different locations in Kenya.

*Location*	*Sampled burrows*	*Tick infested*	*Proportion (%)*
*Kongoni*	14	14	100
*Hippo point*	10	5	50
*Kigio Wildlife conservancy*	12	7	58
*Kedong farm*	10	0	0
*Lewa Wildlife Conservancy*	174	126	72.4

**Table 3:** African Swine Fever Virus positive Tick pools from different locations in Kenya and tested by both qRT-PCR, Conventional PCR and TWIST.

S/N	Field Location	Burrow number	Laboratory Identification number	AFSV qRT-PCR	CT-values	AFSV Conventional PCR	Sanger sequencing	TWIST
1	Kigio	27	28	Positive	28	Positive	AFSV hits	No AFSV hits
2	LWC	9	66	Positive	20	Positive	AFSV hits	AFSV hits
3	LWC	32	72	Positive	20	Positive	AFSV hits	No AFSV hits
4	LWC	32	101	Positive	20	Positive	AFSV hits	No AFSV hits
5	LWC	7	141	Positive	32	Positive	AFSV hits	No AFSV hits
6	LWC	7	151	Positive	20	Positive	AFSV hits	No AFSV hits
7	LWC	9	152	Positive	24	Positive	AFSV hits	AFSV hits
8	LWC	9	156	Positive	32	Positive	AFSV hits	No AFSV hits

### 2.5 ELISA on warthog sera

Serum samples collected from warthogs were analyzed for antibodies against ASFV using a commercial ELISA kit (ID Screen® African Swine Fever Competition, IDvet, Grabels, France) coated with p32 ASFV recombinant protein. The ELISA was performed according to the manufacturer's protocol provided with the kits. The optical density (OD) was read at 450 nm on a microplate reader (ELx808; Biotex). The assay plate was assumed to be valid if the mean value of the positive control OD (ODpc) was less than 0.3 and the mean value of the negative control OD (ODnc) was greater than 0.7. For each sample, the competition percentage (S/N%) was calculated using the formula:

S/N% = (ODsample-ODpc)/(ODnc-ODpc)*100.

Results were interpreted as follows: S/N% ≤ 40 % = positive, 40 % < S/N% < 50 % = doubtful and S/N% ≥ 50 % = negative*.*

### 2.6 Tick processing and DNA extraction

The sampled ticks were washed twice with sterile water to remove contamination, especially soil from the warthog burrows, grass and animal excreta, and rinsed once with 70 % ethanol. Morphological identification based on identification keys (Matthysse, 1987; Okello-Onen, 1999) was done in the laboratory using a stereomicroscope (Leica EZ4D) at x400 magnification. For processing, ticks were pooled into groups of eight according to the burrow and study location. Each tick pool was placed on clean foil, macerated using sterile blades and placed in lysis buffer. The resulting lysate was used for DNA extraction using the ID Gene Spin Universal Extraction kit for DNA purification according to the manufacturer's protocol (IDvet, Grabels, France).

### 2.7 Quantitative real-time PCR

Polymerase chain reaction (PCR) was performed using the ID Gene™ African Swine Fever Virus Triplex Kit (IDvet, Grabels, France) as per the manufacturer's protocol. Every DNA sample taken from the tick pool lysates was subjected to a duplicate PCR procedure with three controls: the negative control from the extraction step, and the positive and negative controls from the kit. The kit is specifically designed to amplify all ASFV DNA genotypes and has both endogenous and exogenous internal controls.

### 2.8 Conventional PCR and Sanger sequencing

PCR was performed using ASFV-specific primers targeting the P72 gene of the ASFV genome (Agüero et al., 2003). Briefly, conventional PCR was performed using the Phusion Green Hot Start II High-Fidelity PCR Master Mix (Thermo Fisher Scientific). For each reaction, 2 μL of template was used together with 10 μL of the 2x Phusion mix, 1.25 μL of both forward (5′-ATGGATACCGAGGGAATAGC-3′) and reverse (5′-CTTACCGATGAAAATGATAC −3′) primers (10 pmol), 0.6 μL of DMSO and 4.9 μL of nuclease-free water, up to a total reaction volume of 20 μL. The PCR products were analysed by gel electrophoresis using 3 % agarose in 1x TAE with GelRed (Biotium Inc. Hayward, CA, US), purified with ExoSAP-IT kit (Thermo Fisher Scientific) and Sanger sequenced at the International Livestock Research Institute, Nairobi, Kenya (ILRI). Sequences were edited and submitted to Genbank under Accession numbers PP421580 –PP421587.

### 2.9 Phylogenetic analysis

Multiple sequence alignment of sequences obtained in this study alongside those of reference strains retrieved from GenBank was performed using MAFFT software v7 (Katoh and Standley, 2013). The aligned sequences were used to reconstruct a maximum likelihood phylogenetic tree (Fig. 4) using the IQ-TREE software v1.6.12 (Nguyen et al., 2015) with 1000 bootstrap replicates. The phylogenetic tree was visualized and annotated using the software FigTree (version 1.4.4, http://tree.bio.ed.ac.uk/software/figtree/)*.*

### 2.10 Pan-Viral panel protocol

To explore the virome of the positive tick pools, we used the Twist Comprehensive Viral Research Panel (CVRP) (PN 103,547, Twist Biosciences, San Francisco, CA, USA). The CVRP is a next-generation sequencing target enrichment protocol specific for viruses (3153 virus sequence references) which has been designed to be applicable within the Illumina TruSeq RNA Library Prep for Enrichment and TruSeq RNA Enrichment workflows (Lwande et al., 2023, 2022). In brief, Illumina TruSeq-compatible libraries were generated from the DNA of the positive tick pools using the Twist Library Preparation EF Kit 2.0 with Enzymatic Fragmentation and the Twist Universal Adapter System (PN 104,207) following the manufacturers protocol. Hybridization capture was performed on the generated libraries using the CVRP and the Twist Target Enrichment Standard Hybridization v1 workflow. Eight indexed sample libraries were pooled equally together, approximately 187.5 ng of each library, and used in a multiplexed 16 h hybridization capture reaction. Following enrichment, libraries were sequenced with 151 bp paired-end reads on the Illumina MiSeq platform, using a MiSeq Reagent Nano Kit, v2 300-cycle kit.

### 2.11 Taxonomic classification and virus genome assembly of metagenomic reads

Paired end reads were filtered by quality and sequencing adapters were removed using fastp. The reads were aligned against the human reference genome GRCh38 and the reads that did not align were filtered out for further analysis. The non-aligned reads were classified using Kaiju (Menzel et al., 2016) and Kraken2 to give a profile of potential virus species in the enriched samples. Sequence reads were also assembled using Megahit (Li et al., 2015) and Trinity (Grabherr et al., 2011) and contigs longer than 1000 bp were kept and polished using Pilon (Walker et al., 2014). The contigs were classified with Blastn against a custom database built with fasta files of viruses, mitochondrial DNA, and mammalian rRNA from GenBank and Refseq.

The remaining contigs were annotated using Prokka (Seemann 2014) and characterized using Checkv (Nayfach et al., 2021) and Virsorter (Roux et al., 2015). Predicted virus sequences were annotated and confirmed using NCBI BLAST+ (Camacho et al., 2009)). To analyze the similarity of the genomic organization of identified ASFVs with other ASFVs, we used Simplot analysis (see also, (34)) [employing R packages ggmsa and gggenes]).

Reads classified as ASFV by Kaiju and Kraken2 were extracted and used as input for MEGAHIT to conduct *de novo* assembly.

## 3 Results

**Fig. 2:**
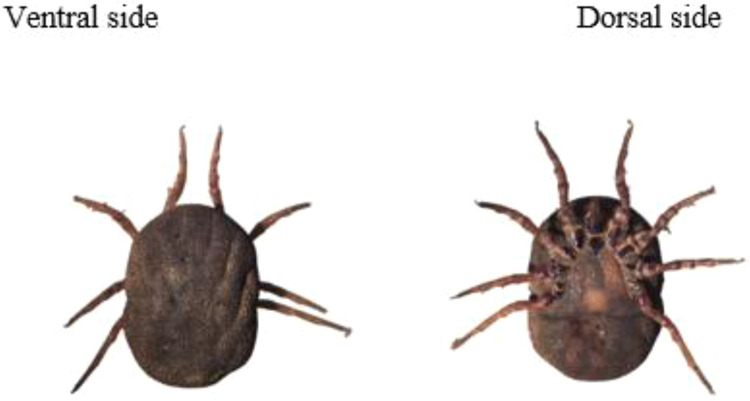
Microscopic images (x400) of the ventral and dorsal side of the *Ornithodoros porcinus* ticks extracted from burrows in Kenya.

**Fig. 3:**
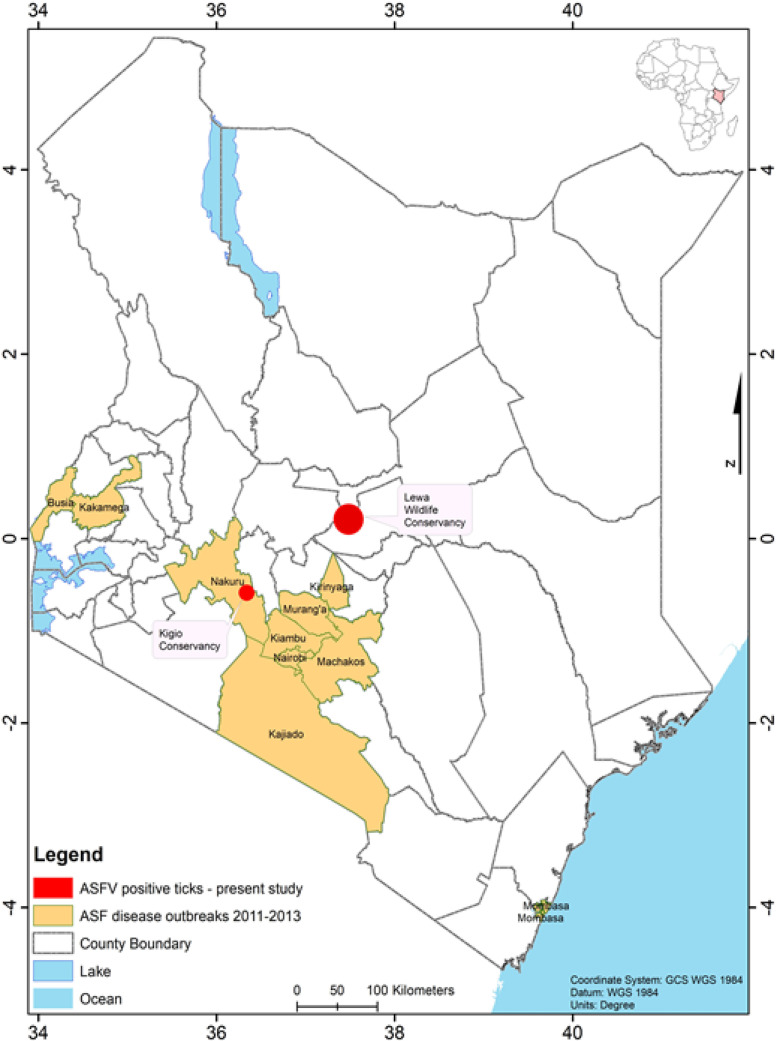
Map of Kenya showing areas with ticks (*Ornithodoros porcinus*) positive with African Swine Fever Virus genotype IX in Kenya. Past ASFV outbreak locations extracted from Onzere et al., 2018.

**Fig. 4:**
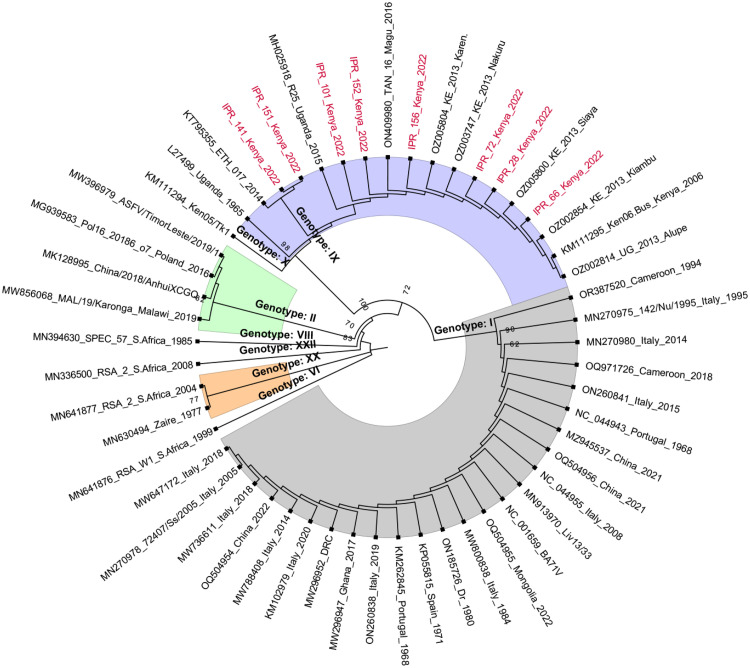
Maximum likelihood phylogenetic tree of ASFV based on nucleotide sequences of partial B646L gene. The tree was generated using IQtree with 1000 bootstrap replicates. Sequences generated in this study are highlighted (red). The numbers at the nodes denote bootstrap values.

We captured and sampled 40 warthogs comprising 21 males and 19 females in eight different locations in Kenya (Table 1). The prevalence of antibodies to ASFV in warthog populations varied across the locations with an overall prevalence of 87.5 %. Warthog samples in Marula and Ol Pejeta Conservancy did not contain antibodies to ASFV. A total number of 228 burrows were investigated and an average of 6 shovel soil scoops were done to collect the ticks. A total number of 2154 ticks were collected, with the highest number collected in Lewa Conservancy. All the ticks were identified morphologically as *Ornithodoros* spp. (Fig. 2). Only three locations, namely, Hippo Point, Kongoni, and Mundui Farm, (Nakuru County), had both ticks and serum samples for testing. Twenty-four (24) out of the 190 tick pools were positive via qPCR whereas 8 pools were positive by conventional PCR. The 8 pools (7 from Lewa Wildlife Conservancy and 1 from Kigio) that were positive via conventional PCR were also positive via qPCR and had CT values ranging between 20 and 30 whereas the remaining 16 tick pools that were positive via qPCR had CT values of above 32 (Table 3). The 8 positive pools by both PCR methods were further subjected to Sanger sequencing where they yielded approximately 303 bases of the ASFV P72 gene. The ASFV in the ticks clustered with genotype IX (Figs. 3 and 4).

### 3.1 Target enrichment protocol

Out of the 8 samples that generated sequences from Sanger sequencing only 2 (from Lewa Wildlife Conservancy) had an ASFV hit by the TWIST target enrichment protocol (200–350 bp in length - Supplementary file 1) but the hits were low (<20 reads) to call. In addition, 60 % of the ticks were identified as *Ornithodoros porcinus*. Other viral sequences identified in the ticks included sequences from *Pandoravirus neocaledonia* and *Choristoneura fumiferana granulovirus*. We also found viral sequences with less than 5 % of reads assigned to Kraken assigned virus strains; Enterobacteria phage p7; Human betaherpesvirus 5 and Human Lymphotropic virus.

## 4 Discussion

This study confirms that warthog populations in Kenya are exposed to ASFV, reflected in the 87.5 % overall seroprevalence in our samples. Such high seroprevalence is likely driven by ASFV-infected ticks since infected adult warthogs excrete too low virus titers to support efficient horizontal transmission (Thomson, 1985; Thomson et al., 1980). Although the viraemic warthog neonates may shed the virus transiently, the developed antibodies are life-long with prevalence reaching up to 100 % among previously infected animals. Ecological factors that drive such high prevalence, occurrence and distribution, especially in Kenya, may include the wide distribution of warthog populations and the presence of competent tick vectors. In the present study, we found co-occurrence of ASFV-exposed warthogs and argasid ticks of the *Ornithodoros* spp. in warthog burrows.

Although *Ornithodoros* spp. are the reservoirs and vectors that maintain the sylvatic cycle, the species composition in each geographical location is often variable and not all biological vectors can withstand virus replication. According to Bakkes et al. (2018), *O.porcinus, O.moubata* and *O.compactus* are a monophyletic group, with *O. phacochoerus, O. porcinus*, and *O.waterbergensis* playing key roles in the sylvatic cycle (Bakkes et al., 2018). Even though the ranges of *O. porcinus* and *O. moubata* overlap in Kenya (Jori et al., 2023), the virus may behave differently depending on the tick species. Within the warthog landscape, a burrow, often dug by other mammals, is a suitable microhabitat that nurtures the nidiculous and photophobic *Ornithodoros* spp. ticks. However, these ticks can also occur outside on the burrow openings, or in other warthog resting spots such as culverts and shaded vegetation. In the present study, the proportion of tick-infested burrows was variable and ranged between 50 and 100 %, except in Kedong Ranch where no tick was recovered from the burrows. It is interesting that Kedong, the cradle for ASFV and the place where the first outbreak was reported over 100 years ago (Montgomery, 1921), lacked soft ticks in burrows. Reported proportions of burrows with infested ticks range between 30 % - 88 % (Jori et al., 2023), which is consistent with our study. The farrowing seasonality of warthogs and time spent with neonates in the burrow is suggested to influence the presence/absence of ticks and the proportion of ticks per burrow as well as the number of virus-positive ticks (Jori et al., 2023; Thomson, 1985). In this study, the proportion of soft ticks per burrow ranged from 2.9 % to 13.9 %, which may be driven by the tick intrinsic characteristics, host preferences or burrow-use dynamics by other mammals, such as the Aardvark (*Orycteropus afer*) and Porcupines (*Hystrix cristata*) (Peirce, 1974). Apart from the effects of multi-species use of the burrows on the dynamics of *Ornithodoros* spp., other mammals such as Aardvark may scatter the ticks out of the burrow as they re-dig the burrows. In addition, the burrow-use dynamics by Multiple species are likely to influence the tick microbial communities.

Our results showed that the *O. porcinus* tick populations in Lewa Wildlife Conservancy and Kigio Conservancy were infected with ASFV genotype IX that phylogenetically clusters with ASFV previously associated with outbreaks in domestic pigs in western Kenya and eastern Uganda (Onzere et al., 2018). The outbreaks in 2011 and 2013 in domestic pigs in Kenya were due to ASFV genotype IX which occurred in 11 counties, with a concentration in the Central and Rift Valley counties (Onzere et al., 2018). The detection of ASFV genotype IX in ticks at the Kigio Conservancy (Nakuru County), an area that overlaps with foci of previous domestic pig outbreaks, suggests co-circulation of the same virus genotype in the sylvatic and domestic pig cycles with *O. porcinus* as the main reservoir and vector in the sylvatic cycle. Previous studies have detected genotype X in soft ticks in Kenya (Gallardo et al., 2011), which suggests that ASFV genotypes IX and X circulate among ticks, warthogs and domestic pigs (Jori et al., 2023; Lubisi et al., 2005; Plowright et al., 1969). A limitation of this study is that we could not analyse ASFV genotypes in the sampled warthogs.

Moreover, the target enrichment protocol failed to yield the entire ASFV virus genome as expected, but instead only a few reads of the virus from two of the 8 positive Sanger-sequenced samples. Other viruses detected were associated with invertebrates, animals and humans; *Pandoravirus neocaledonia; Choristoneura fumiferana granulovirus*; Enterobacteria phage p7; Human Lymphotropic virus and Human betaherpesvirus 5. However, since only occasional reads were identified as these viruses, it is possible that the results could be false positives. There were also low levels of fragments of other viral DNA/RNA, but the levels were too low to be able to draw any conclusions. The virome of ticks needs to be further investigated and methods could be improved to increase the sensitivity, for example by more effective depletion of host DNA.

The presence and role of *Pandoravirus neocaledonia* (Pandoraviridae family) in the tick is previously unknown, though it was first isolated from the brackish water around a mangrove (New Caledonia) (Legendre et al., 2019). Further, the presence of *Choristoneura fumiferana granulovirus* in soft ticks (*O. porcinus,* is the first record as previously it was recorded as an abundant commensal virus of hard ticks (*Ixodes* spp. and *Haemaphysalis* spp.) in China (43). *Granuloviruses* (GV) are members of the family *Baculoviridae* that are specific to insects in the Orders of *Diptera, Hymenoptera,* and *Lepidoptera* and their presence in other invertebrates is refuted or disputed, but they are important biopesticides used in sub-Saharan Africa (Gilligan, 2014; Moore and Jukes, 2023). *Enterobacteria phage 7* is thought to be part of the enterobacterial symbiont of the common bed bugs (*Cimex lectularius*) (Sheele et al., 2023) and here reported for the first time in *O. porcinus* ticks.

The twist enrichment protocol on the ASFV-positive soft ticks yielded a few reads aligning with human pathogens. Human betaherpesvirus 5, also called human cytomegalovirus (HCMV) belongs to the family Herpesviridae or herpesviruses; Human Lymphotropic viruses are a family of retroviruses which cause chronic life-long infections in people. acquisition of these human pathogens by *O.porcinus* could be through blood sources from humans, which is rare in non-domestic settings as in the current study. Since we do not have whole genomes of the viruses it could also be sequences from closely related herpesvirus from another mammal for example a primate. All of this could also be due to reads being incorrectly classified by Kaiju and Kraken2. We have also noticed that some virus strains handled in the lab, *e.g. hantaviruses* were detected at low level in some of the samples. This is most likely due to contamination, either in the lab or in the Illumina instrument.

The plausible explanation is on burrow-use dynamics leading to tick-human contact, such as; (1) a warthog dashes out of the burrow, *O. porcinus* ticks may dislodge late and get dropped at the burrow entrance/surrounding – especially the *Ornithodoros* nymphs (Boomker et al., 1991; Horak et al., 1988, 1983); or (2) another mammalian-burrow user modifies the burrow by digging out the surface soil inside the burrow and scatter some ticks on the burrow surrounding; or (3) the herders sit on the burrow mound as they are the only cleared raised ground that gives them a vantage wide view to watch their grazing livestock.

In conclusion, our results provide evidence of the sylvatic cycle of genotype IX ASFV, which through *O. porcinus* tick bites has resulted in high exposure in adult common warthogs. The ecology of *Ornithodoros* spp. and burrow-use dynamics are complex and more studies are needed to understand the dynamics of these ticks in the sylvatic cycle and specifically in the spread of ASFV at the wild suid-domestic pig interface. The *Ornithodoros* spp. seems to have a rich virome, which has not been explored but could be exploited towards ASF control strategy.

## 5 Future prospects

Due to limited funds, we could attempt extensive diagnostics such as PCR and sequencing of warthog samples and culture of PCR-positive tick samples. Such investigations would be useful for potential identification of the circulating genotypes and evolutionary tracking. The target enrichment protocol via TWIST is an expensive and tedious technique which we explored knowing that it targets mostly RNA viruses and therefore the detection of ASFV warrants the development of a more comprehensive panel that targets DNA viruses. The field samples from this study could be used for further investigation of the mechanisms in which ASFV induces its effect on diverse hosts and tick vectors.

## Funding

This project was supported by the Swedish Research Council (Vetenskapsrådet), Research Network Grant with a focus Swedish Research Links, Registration number 2021–05307. Basic Science-Oriented Biotechnology Research Grant at the Faculty of Medicine, Umeå University (grant 2022–2023), Formas Grant (2020–01056) and SciLifeLab Pandemic Laboratory Preparedness (PLP) program.

## Ethics approval and consent to participate

The study protocol was approved by the Wildlife Research and Training Institute (WRTI), the state agency mandated to issue research authority in the wildlife sector, under the permit number WRTI-0143–01–22. The capture and sampling were carried out by the immobilization protocol of the Kenya Wildlife Service

## Consent for publication

Not applicable.

## CRediT authorship contribution statement

**Vincent Obanda:** Writing – review & editing, Writing – original draft, Visualization, Validation, Supervision, Software, Resources, Methodology, Investigation, Funding acquisition, Formal analysis, Data curation, Conceptualization. **Mercy Akinyi:** Writing – review & editing, Visualization, Validation, Methodology, Investigation, Formal analysis, Data curation. **Edward King'ori:** Writing – review & editing, Visualization, Validation, Methodology, Investigation, Data curation. **Ruth Nyakundi:** Writing – review & editing, Visualization, Validation, Methodology, Investigation, Formal analysis, Data curation. **Griphin Ochola:** Writing – review & editing, Methodology. **Purity Oreng:** Writing – review & editing, Methodology, Investigation. **Kevin Mugambi:** Writing – review & editing, Methodology, Investigation. **Grace Mwihaki Waiguchu:** Writing – review & editing, Visualization, Validation, Methodology, Investigation. **Mary Chege:** Writing – review & editing, Validation, Methodology, Investigation, Data curation. **William Rosenbaum:** Writing – review & editing, Visualization, Validation, Software, Methodology, Investigation, Data curation. **Erik Bovinder Ylitalo:** Writing – review & editing, Methodology, Investigation. **Anne Tuiskunen Bäck:** Writing – review & editing, Visualization, Validation, Supervision, Resources, Methodology, Investigation. **Lisa Pettersson:** Writing – review & editing, Visualization, Validation, Resources. **Opanda Silvanos Mukunzi:** Writing – review & editing, Visualization, Validation, Software. **Bernard Agwanda:** Writing – review & editing, Visualization, Validation, Supervision, Methodology, Investigation, Formal analysis, Data curation, Conceptualization. **Susanna Stenberg-Lewerin:** Writing – review & editing, Visualization, Validation, Supervision, Investigation, Conceptualization. **Olivia Wesula Lwande:** Writing – review & editing, Writing – original draft, Visualization, Validation, Supervision, Software, Resources, Project administration, Methodology, Investigation, Funding acquisition, Formal analysis, Data curation, Conceptualization.

## Declaration of competing interest

The authors declare no competing interests.

## Data Availability

Data will be made available on request.

## Appendix Supplementary materials

Supplementary Figure 1 Evidence of raw reads of ASFV generated from tick species (*Ornithodoros porcinus*) via the target enrichment Image, application 1
