# Supplementary material for: Epidemiology and ecology of the sylvatic cycle of African Swine Fever Virus in Kenya
Source: Virus Res. 2024 Jul 19;348:199434. doi: 10.1016/j.virusres.2024.199434 (PMC11325071; doi:10.1016/j.virusres.2024.199434)
Supplement: Supplementary Figure 1 — Evidence of raw reads of ASFV generated from tick species (Ornithodoros porcinus) via the target enrichment [file mmc1.pdf]

Contigs

Which samples had ASFV contigs?

```
blastn %>%
  filter(str_detect(match_name, "swine")) %>%
  select(sample_id, match_name, read_len) %>%
  knitr::kable()
```

sample_id	match_name	read_len
OWL2_S7_R	African swine fever virus strain Ken05/Tk1, complete genome	439
OWL6_S3_R	African swine fever virus isolate Kenya 1950, complete genome	430

- Sample 2 and 6
  - Same samples with the most reads classfied by kraken and kaiju

Which samples had contigs of Ornithodoros porcinus?

```
blastn %>%
  filter(str_detect(match_name, "Ornithodoros porcinus")) %>%
  select(sample_id, match_name, read_len) %>%
  knitr::kable()
```

sample_id	match_name	read_len
OWL2_S7_R	Ornithodoros porcinus mitochondrion, complete genome	954
OWL2_S7_R	Ornithodoros porcinus mitochondrion, complete genome	412
OWL8_S1_R	Ornithodoros porcinus mitochondrion, complete genome	700
OWL8_S1_R	Ornithodoros porcinus mitochondrion, complete genome	460
OWL1_S8_R	Ornithodoros porcinus mitochondrion, complete genome	458
OWL3_S6_R	Ornithodoros porcinus mitochondrion, complete genome	676
OWL3_S6_R	Ornithodoros porcinus mitochondrion, complete genome	559
OWL7_S2_R	Ornithodoros porcinus mitochondrion, complete genome	446
OWL5_S4_R	Ornithodoros porcinus mitochondrion, complete genome	427

All samples except sample 4, and some samples had more than one.
